# Modification of Yellow Fever by Preceding Diseased States of the System

**Published:** 1873-06

**Authors:** Joseph Jones

**Affiliations:** Professor of Chemistry and Clinical Medicine, Medical Department University of Louisiana, Visiting Physician of Charity Hospital, New Orleans


### MODIFICATION OF THE PHENOMENA OF YELLOW FEVER BY PRECEDING DISEASED STATES OF THE SYSTEM.

BY JOSEPH JONES, M.D.,

*Professor of Chemistry and Clinical Medicine, Medical Department University of Louisiana, Visiting Physician of Charity Hospital, New Orleans.*

The most potent cause of derangements in those who reside within the yellow-fever zone is the action of malaria, which not only manifests its effects in the causation of intermittent, remittent, pernicious and hemorrhagic paroxysmal fevers, which differ materially in their origin and symptoms and pathology from yellow fever, but also without the active manifestation of these forms of fever, in the slow destruction of the colored corpuscles, derangements of the liver, enlargement of the spleen, attended with a pale, sallow, sickly hue, infiltration of the cellular tissue, dyspnœa, palpitations and depressions of the muscular and nervous forces.

While those who have been subjected, for long periods, to the action of malaria appear to be less liable to yellow-fever, it is, however, true that the progress of this disease is modified by the changes induced in the blood and organs by the preceding action of the malarial poison; and the lesions after death, from yellow fever, differ to a certain extent from those observed in subjects freshly arrived from cold climates, without being previously subjected to the action of the marsh miasm. And in every epidemic of yellow-fever, the malarial influence is so powerful in most of the cities of the tropical and sub-tropical zone, that it is never entirely suspended, and not only in many cases induces characteristic changes before the specific action of the yellow fever poison, but also is frequently engrafted upon the weakened convalescents from yellow fever, thus altering the farther progress of such cases, and inducing changes in the organs, entirely different from those characteristic of yellow fever. In this continuous and preceding action of the malarial poison, and in the frequent intermingling of the two diseases, we have an explanation of the apparently contradictory statements of observers as to the characteristic symptoms and lesions of yellow fever. It is evident, therefore, that no observer is competent to the elucidation of the pathology of yellow fever who is not at the same time familiar with the changes induced by the various forms of paludal fever and malarial poisoning.

If we accept without reserve the doctrine advocated by John Hunter,* and ably supported by Joseph Adams,† that "No two actions can take place in the same constitution, or in the same part, at one and the same time; no two different fevers can exist in the same constitution—no two local diseases in the same part at the same time," then the question of the modification of malarial fever by yellow-fever, and of the engrafting of the one upon the other, must be definitely settled in the negative.

We have elsewhere shown by an extended discussion‡ of this question, that whilst, where two poisons, representing two distinct exanthemic diseases, act simultaneously upon the human being, the most obvious pathological phenomena excited by the poisons will not occur simultaneously, but in succession, the one poison retarding the action of the other—the one producing its cycle of changes whilst the other remains "dormant," as it were, during the action of the other, and immediately after the changes induced by the first cease, inducing in turn its own distinct effects. At the same time it must be admitted that the character and course of these specific eruptive diseases are greatly modified by such altered states of the constitution as exist in scurvy, scrofula, and secondary syphilis.

* Works of John Hunter, London edition: Vol. ii, Treatise on the Venereal Disease, p. 132; vol. iii, Treatise on the Blood, pp. 3–5; vol. i, Principles of Surgery, pp. 312–313.

† Observations on Morbid Poisons, Chronic and Acute, London, 1807, pp. 21–23.

‡ Researches on Spurious Vaccination, or the Abnormal Phenomena Accompanying and Following Vaccination in the Confederate Army, during the recent American Civil War—1861–1865.

In that class of diseases represented by constitutional syphilis, scurvy and malarial poisoning, the blood is at fault, the nutrition is perverted, and the course and products of diseased actions are correspondingly modified.

During the recent war I embraced an opportunity of observing the effects of the engrafting of small-pox upon systems broken down by exposure, privation, and the exhaustive effects of hospital gangrene and pyæmia.

In the month of September, 1864, small-pox spread from the ward devoted to the treatment of the disease to the Empire Hospital, which had been filled with cases of hospital gangrene and pyæmia, gathered from the general hospitals attached to the Confederate army operating in and around Atlanta. The small-pox ward was situated in a pine-grove about three hundred yards in the rear of the gangrene hospital, and, without doubt, communication was kept up between the different wards. Several of the nurses of the gangrene hospital were first attacked, and the disease appeared to have been communicated from them to the patients under their charge, who were suffering with hospital gangrene and pyæmia.

The following case arrested my attention as clearly illustrating the engrafting of small-pox upon a system reduced by hospital gangrene:

James S. Johnson, company K, 19th Alabama regiment, age 21; wounded at the siege of Atlanta, July 22, 1864; gunshot wound of right leg, which was amputated on the field of battle at the lower third of the thigh. Hospital gangrene attacked the stump, and the patient was admitted to the Empire Hospital on the 22d of September.

Nitric acid arrested the progress of the gangrene for a time, but it returned again, and the strength of the patient was gradually reduced by the absorption of the gangrenous matter, and from an exhausting diarrhœa.

On the 2d of November, whilst the patient was exceedingly feeble and nervous, and manifested the symptoms of pyæmia—viz: chills, icturus, and vomiting of dark green matters—the eruption of variola made its appearance. The pustules, which were in considerable numbers, but small in size, progressed regularly, and on the 6th of November presented all the characters of the true variolous eruption, with the round form and umbilicated center. The appearance was similar in all respects to the pustules of patients not suffering with gangrene or pyæmia. This patient had a good vaccine scar, and the smallness of the pustules appeared to be due to the fact that the system was partially protected by vaccination.

The patient died during the night of the 6th, apparently from the effects of the gangrene, pyæmia, and exhausting diarrhœa.

Not one of the pustules in the preceding case presented any appearance of hospital gangrene; and, as far as I was able to learn, both during my investigation and subsequently, no case occurred in the gangrene hospital in which humid gangrene attacked the eruption of small-pox or variola.

It was clearly established that small-pox would attack patients suffering with gangrene, and even pyæmia, when they were greatly reduced in flesh and strength.

In an interesting case which occurred in my private practice, immediately after the close of the war, acute dysentery, of a severe and painful character, was superseded by typhoid fever, which was well marked in its symptoms and progress by low-muttering-delirium, diarrhœa, tympanitis, lenticular, rose-colored spots, and painful enlargement of the parotid glands. As soon as well marked typhoid symptoms were manifested, all the symptoms of acute dysentery vanished, and the patient passed, without pain or straining, the ordinary loose (*pea-soup*) stools of typhoid fever, and the smallest doses of purgative medicines, either mercurial or saline, acted with great and dangerous violence.

This patient passed safely through the typhoid fever, and even the parotid glands subsided without suppuration; but in the third week after the appearance of typhoid fever, when the patient was apparently doing well, the dysentery returned, with severe straining, and bloody and mucoid discharges, attended with severe pain. The copious (*pea-soup*) evacuations of typhoid fever were completely superseded by the small, frequent, bloody mucoid discharges of acute dysentery, and the patient died in consequence of the return of the original disease.

I have also observed that typhoid fever was often engrafted upcn malarial fever, amongst the Confederatc troops serving in malarious districts, and that during its active stages it presented the usual continuous fever, with cerebral disturbance, agitation of the muscles, tympanitis, diarrhœa, and lenticular rose-colored spots. As soon as the typhoid fever was established, all signs of periodicity disappeared, and the torpid liver of malarial fever was exhibited; the sallow hue of malarial fever became clear from the increased action of the liver; the torpid bowels became loose and tympanitic; and after the disappearance of the symptoms characteristic of typhoid fever, the original malarious paroxysmal fever reappeared with its cold and hot stages recurring at regular intervals, and inducing torpor of the liver and bowels and the sallow malarial hue.

As far as my observation extends, in like manner, when yellow fever is engrafted upon a system previously under the influence of the malarial poison, it establishes its own peculiar train of symptoms, which are distinct from those of malarial fever, and may be clearly recognised; when, however, the cycle of changes excited by the yellow-fever poison, has been completed, then the malarial poison may again excite its characteristic recurring paroxysms.

New Orleans, June 4, 1873.

